# Supplementary figures and images for: Integrating questionnaire measures for transdiagnostic psychiatric phenotyping using word2vec
Source: PLoS One. 2020 Apr 3;15(4):e0230663. doi: 10.1371/journal.pone.0230663 (PMC7122719; doi:10.1371/journal.pone.0230663)

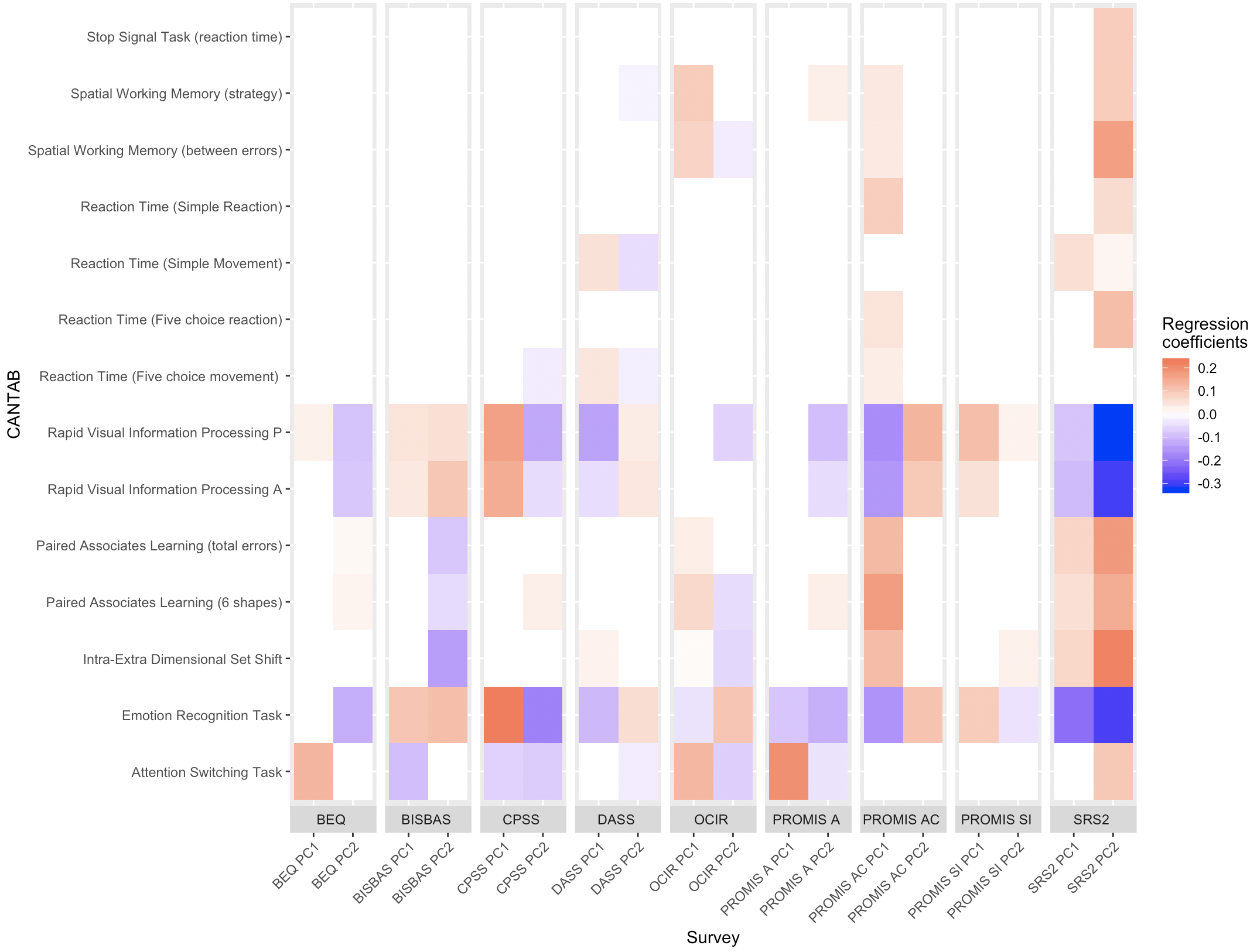

Supplement: S1 Fig — (TIFF) [file pone.0230663.s001.tiff]

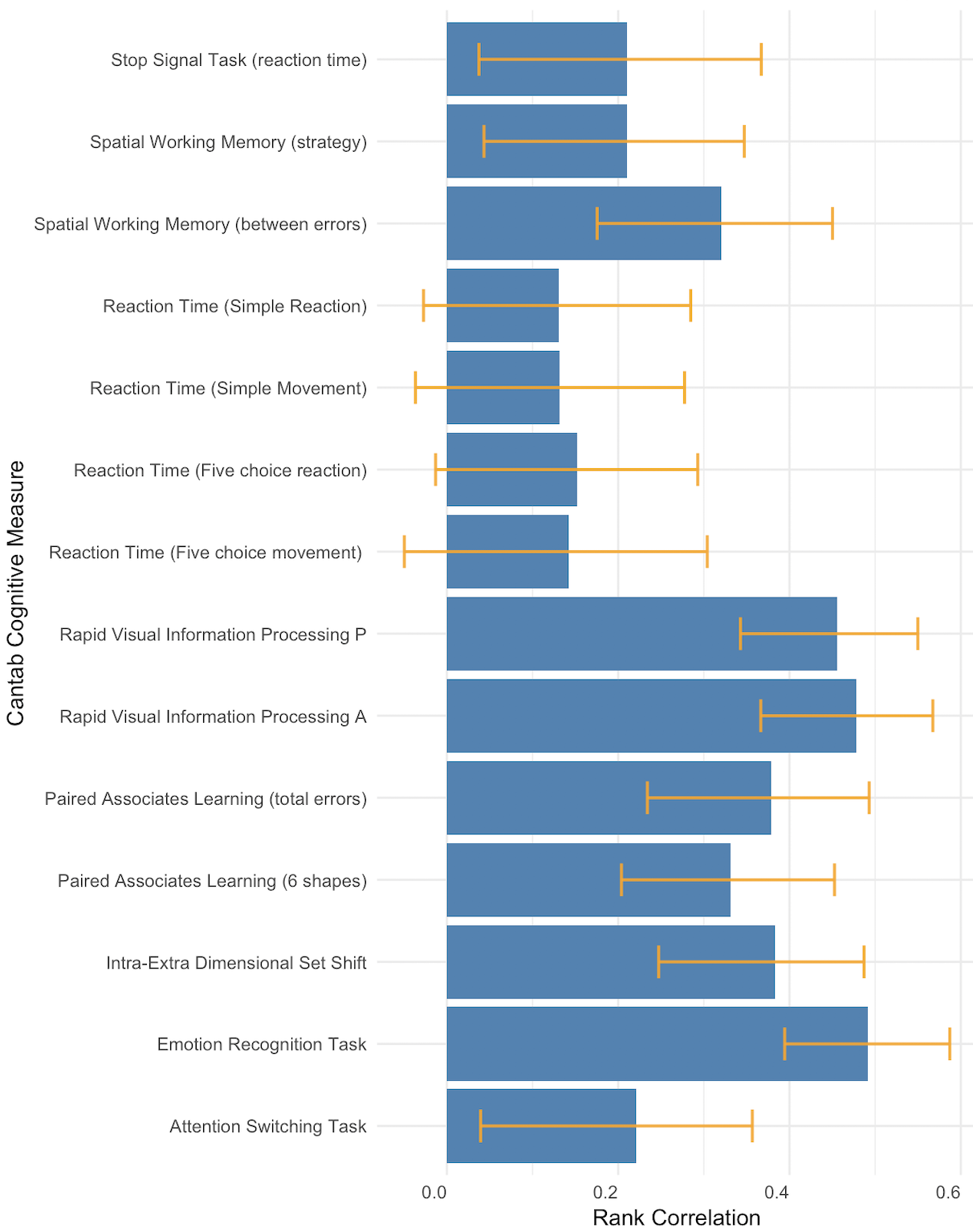

Supplement: S2 Fig — Plot shows correlation over a 10-fold cross-validated regression for 500 bootstrapped samples. 95% bootstrap confidence intervals are shown in orange. (TIFF) [file pone.0230663.s002.tiff]
