# Supplementary material for: Integrating questionnaire measures for transdiagnostic psychiatric phenotyping using word2vec
Source: PLoS One. 2020 Apr 3;15(4):e0230663. doi: 10.1371/journal.pone.0230663 (PMC7122719; doi:10.1371/journal.pone.0230663)
Supplement: Attachment — Submitted filename: Rebuttal_2020_Jan24_rp_ab.docx [file pone.0230663.s003.docx]

**Dear Editor,**

Thank you for the careful review of our manuscript, "Integrating questionnaire measures for transdiagnostic psychiatric phenotyping using word2vec". Below, we summarize the reviewers' critiques and our revisions.

We appreciate the opportunity to revise our work.

**Reviewer #1**

**The major limitation in this paper is that the stated aim, to “validate the clinical utility of the embedding scores,” is never directly tested and instead there are just multiple transformations of scores into high and then low dimensional spaces, resulting ultimately in models which can distinguish psychiatric patients from healthy controls using nine assessments of various clinical symptoms.**

1) One key aspect lacking from these multiple tests and manipulations is no direct test of added benefit from applying word embeddings to the scores. Could one not simply take the multidimensional data space of the scales themselves and subject this data space to dimensional reduction? If anything is gained by the additional step of training the word embedding vectors, this is unclear from the paper as written.

Response: Thank you for the constructive comment. We believe there are two benefits of integrating word embeddings to the questionnaire scores, a quantitative and a qualitative one.

With respect to the quantitative advantage, we thank the reviewer for the observation that there was no hard evidence that points to a benefit. We now demonstrate this benefit by including the classification performance of the unsupervised clustering and supervised regression methods using the questionnaire raw ordinal scores with the same dimensionality reduction techniques as a benchmark.

Results shown in Table 1 in the manuscript (and below) suggest that incorporating embeddings into the classification generally improves the accuracy over the classification obtained directly based on the ordinal scores with substantial improvements for some cases. The most substantial improvement is observed using the first canonical correlation variable (CCV) obtained from the scores and DSM-V Self-Rated Symptom Measure. Permutation tests where used to test the difference in accuracy and AUC bootstrap means between the embedding scores and raw response scores. For example, The AUC for unsupervised classification using CCV, increases from 0.75 , 95% CI (0.58, 0.93) or 0.76 , 95% CI (0.59, 0.94) to 0.84, 95% CI (0.71 0.92) or 0.84, 95% CI (0.73 0.91) for cases vs. controls+super controls (p-val<0.001), for cases+controls vs. supercontrols (p-val<0.001). We have now included these additional comparisons in the manuscript.

Table 1. Accuracy (ACC), AUC, false positive rate (FPR), and false negative rate (FNR) are obtained using supervised (logistic regression) and unsupervised (Gaussian mixture modeling) classification. All metrics are based on a probability of 0.5 classification

We would like to emphasize that classification performance is shown as one quantitative validation that demonstrates the utility of the proposed embedding-scores as summaries of patient profiles. The utility of these scores, however, is not limited to the specific classification considered in our validation study. We have now further included discussions on how these embedding-based patient profiling can be used in alternative contexts; for example, we used them to infer patients scores for specific RDoC domains.

Regarding the *qualitative* advantage of adding word embeddings, in line with explicit NIMH priorities, our method provides dimensional rather than categorical approaches to disease. As we state in the discussion, we try to address two challenges that arise in survey scoring as currently performed: 1) questions which might be of different relevance towards contributing diagnostic information are equally weighted and counted for phenotyping, 2) discrete scores impose an artificial threshold for disease severity, leading to a coarse measure of patient behavior. Page 16 and 17 describe in detail why embedding scores are useful to address these 2 challenges. We provide further detail in our responses below.

2) It is also unclear exactly how transforming a 42-item questionnaire (like the DASS) into a 500-dimensional vector provides additional information, and it appears as though every questionnaire was subjected to this same high dimensional representation.

Response: We agree that 500 is not low per se, although it is significantly lower than the number of unique words appearing in the survey. A substantial benefit of incorporating embedding is to leverage semantic similarities between different words/contexts which is not possible with the ordinal scores. As mentioned above, such an approach also enables us to create RDoC scores for individual patients based on their questionnaire data. To address the high-dimensional word embeddings, we further perform dimension reduction through principle component, and canonical correlation analysis of the questionnaire level embedding vectors and carry out subsequent analysis using the leading 1, 2 PCs and 1 CCV per questionnaire, this allows us to summarize scores from all questionnaires into relatively low dimensional vectors: 9, 18 and 9 dimensions respectively.

3) We are given no examples of how the word embedding representations map onto various RDoC domains, DSM diagnoses, or symptoms.

Response: We thank the reviewer for the very constructive comments and have now added a method for constructing RDoC scores based on the questionnaire data for each patient as detailed in the revised manuscript. In brief, we calculate the RDoC scores based on cosine similarity between a reference RDoC domain vector and the patient RDoC domain specific embedding vector. These RDoC domain embedding vectors are based on weighted averages of the embedding vectors for the seed words representative of the RDoC domains as given in McCoy et. al. 2018. These RDoC scores are then validated by measuring their rank correlation to the CANTAB neurocognitive scores. Results are shown in Figure 4 in the manuscript (and below). Many of these correlations are robust, suggesting the ability of these RDoC domain scores to capture neurocognitive function. As expected, the Cognitive Systems domain is highly correlated to multiple CANTAB scores.

Figure 4: RDoC score Rank Correlation Heatmap: we calculate the rank correlation between the selected CANTAB cognitive score metrics and embedding-based derived RDoC scores. High correlation is shown in warm colors. Significance is shown with stars: 0.1*, 0.05**, and 0.01 ***.

Figure 4. RDoC score Rank Correlation Heatmap: we calculate the rank correlation between the selected CANTAB cognitive score metrics and embedding-based derived RDoC scores. High correlation is shown in warm colors. Significance is shown with stars: 0.1*, 0.05**

4) In the discussion, there are statements that this word embedding representation “captures each question’s meaning as opposed to just the ordinal response,” however, this quality of the analysis is not demonstrated at all. Furthermore, since the questions are uniform between participants, how does this technique “quantify how the language is perceived by the responder” when every single participant will answer with an ordinal response.

Response: We agree with the reviewer that this statement was overly expansive. We have rephrased the paragraph where we state that our approach can “*quantify how the language is perceived by the responder*” in the discussion section. We mean to say, as previously stated in the responses, that embedding based approach enables us to leverage similarities/relatedness of different questions when summarizing patient profile. Leveraging embeddings also allows us to easily extract different components of the questions to reflect more specific aspects such as RDoC domain, which is not feasible using raw questionnaire scores alone.

In the introduction and discussion, there is mention of the five RDoC domains, but there is no explicit mention or investigation of any of the representations in terms of these domains, although I’m sure some could be implied.

Response: We thank the reviewer for this comment. We have now included a method for calculating RDoC domain scores based on the questionnaires and embeddings as discussed above.

5) Throughout the paper there is mention that these representations were compared to Axis 1 diagnoses, but the actual analyses seem to reduce this to super controls having no lifetime history of Axis 1 Dx, controls having one lifetime Axis 1 that is not MDD, bipolar, schizophrenia or schizoaffective and “cases” having current diagnoses of MDD, bipolar, schizophrenia or schizoaffective. This appears to provide limited clinical utility.

We have ensured that we do not assert utility in distinguishing among diagnoses; we explicitly adopt a transdiagnostic approach in which dimensional illness features are the focus, rather than disease categories. We have clarified that the point of these comparisons is simply to show differences between symptomatic and asymptomatic individuals, complementing dimensional analyses.

References:

McCoy, T. H., Yu, S., Hart, K. L., Castro, V. M., Brown, H. E., Rosenquist, J. N., Doyle A.E., Vuijk P.J., Cai T., Perlis, R. H. (2018). High Throughput Phenotyping for Dimensional Psychopathology in Electronic Health Records. Biological Psychiatry, 83(12), 997–1004. doi: 10.1016/j.biopsych.2018.01.011

**Reviewer #2**

**First of all, I would like to congratulate the authors for their effort to establish a transdiagnostic tool. Hypothesis is well defined, the method is mind-provoking, results of the study might be an important step for the development of future artificial intelligence technology that would allow to screen general psychiatric symptoms apart from the descriptive categorical diagnosis.**

Response: Thank you for these positive comments.

1) Authors report that symptom dimensions were chosen considering the RDOC. However, it is difficult to conclude that RDOC describes all prominent dimensions of general psychiatric pathology. Besides, it is still concurrent with the most recent version of classical descriptive diagnostic categories. Therefore, authors should indicate other evidence for the reason of RDOC choice.

Response: We have clarified our rationale for focusing on RDoC domains.

2) It is interesting to see that cognition was chosen as another domain for this proposed diagnostic tool and CANTAB was used for the evaluation. That would be better to read the reasons in detail.

Response: We added further detail regarding our use of the CANTAB battery here.

3) Impact of the treatment and also disease related factors on this instrument should be discussed in different aspects for each disorder considering different disease and treatment characteristics.

Response: In the discussion, we recognize the importance of considering treatment effects in future work.

4) A couple sentences should be added to conclusion in terms of future predictions and contributions to the field

Response: We have expanded our discussion to address potential next step investigations.
